# The “Flu” Poem

**Published:** 1919-01

**Authors:** 


					﻿£iii>iiiitiiiti|iiiiiiiiiiiiiiiiiiniiiitmiiiiiiiiiiitit(iiiiitiuimiiiiiiiiiijiiitiiiiiiiiiiiitiitiiiiiiiiiii!iiiiiiiiiiiiiiiiiiniiiiiiiiiiiiiiiiiiiiiiiitiimiiiiiiiiiiittiiiiiitiiitiiiiiiiiiiiiimiiiiiiiiii!iiiitiiiiiiiiiiiiiiiitt
I MEDICAL MISCELLANY |
niiiiiiiiijiiiiiiiiiiiiiiiiiiiiiiiiiiiiiiiiiiiiiiiiiiiiiiiiiiiiiiiiiiiiiiiiiiiiiiiiiiiii'iiiniiiiiiiiiiiiiiiiiiiiiiiiiiiiiiiiiiiiiiiiiiiiiiiiiiiiiiiiiiiiiiiiiiiiiiiiiiiiiiiiiiiiiiiiiiiiiiiiiiiiiiiniiiiiiiitiitiiiiiiiiiiiiiiiiiiiiiiiiiiin
The Flu.
When your back is broke and your eyes are blurred,
And your shin bones knock and your tongue is furred,
And your tonsils squeak and your hair gets dry,
And you’re doggone sure you’re going to die,
But you ’re skeered you won’t and afraid you will—
Just drag to bed and have your chill,
And pray to the Lord to see you through
For you’ve got the flu, Boy,
You’ve got the flu.
When your toes curl up and your belt goes flat,
And you’re twice as’means as a Thomas cat,
And life is one long and dismal curse,
And your food all tastes like a hard-boiled hearse,
When your lattice aches and your head’s a-buzz?
And nothing is as it ever was,
Here are my sad regrets to you—
You’ve got the flu, Boy,
You’ve got the flu.
What is it like, this Spanish flu?
Ask me, Brother, for, I’ve-been through.
It is misery out of sheer despair;
It pulls your teeth and curls your hair,
It thins your blood and brays your bones,
And fills your craw with moans and groans,
And sometimes, may be, you get well,
Some call it flu.—I call it HELL.
—Anonymus.
				

